# Long-term effects of bilateral pallidal deep brain stimulation in dystonia: a follow-up between 8 and 16 years

**DOI:** 10.1007/s00415-020-09745-z

**Published:** 2020-02-13

**Authors:** P. Krause, S. Völzmann, S. Ewert, A. Kupsch, G. H. Schneider, Andrea A. Kühn

**Affiliations:** 1 Movement Disorder and Neuromodulation Unit, Department of Neurology, Charité, University Medicine Berlin, Campus Mitte, Charitéplatz 1, 10117 Berlin, Germany; 2 Department of Neurosurgery, Charité, University Medicine Berlin, Campus Mitte, Berlin, Germany; 3 Department of Neurology and Stereotactic Neurosurgery, University Medicine of Magdeburg, Magdeburg, Germany

**Keywords:** Dystonia, Pallidal DBS, Long-term effects, DBS and quality of life

## Abstract

**Objective:**

Observational study to evaluate the long-term motor and non-motor effects of deep brain stimulation (DBS) of the globus pallidus internus (GPi) on medically refractory dystonia.

**Background:**

Dystonia is a chronic disease affecting mainly young patients with a regular life expectancy and lifelong need for therapy. Pallidal DBS is an established treatment for severe isolated dystonia but long-term data are sparse.

**Methods:**

We considered 36 consecutive patients with isolated generalized (*n* = 14) and cervical/segmental (*n* = 22) dystonia operated at Charité-University Hospital between 2000 and 2007 in a retrospective analysis for long-term outcome of pallidal DBS. In 19 of these patients, we could analyze dystonic symptoms and disability rated by the Burke–Fahn–Marsden Dystonia Rating scale (BFMDRS) at baseline, short-term (ST-FU, range 3–36 months) and long-term follow-up (LT-FU, range 93–197 months). Quality of life and mood were evaluated using the SF36 and Beck Depression Index (BDI) questionnaires.

**Results:**

Patients reached an improvement in motor symptoms of 63.8 ± 5.7% (mean ± SE) at ST-FU and 67.9 ± 6.1% at LT-FU. Moreover, a significant and stable reduction in disability was shown following DBS (54.2 ± 9.4% at ST-FU and 53.8 ± 9.2% at LT-FU). BDI and SF36 had improved by 40% and 23%, respectively, at LT-FU (*n* = 14). Stimulation-induced adverse events included swallowing difficulties, dysarthria, and bradykinesia. Pulse generator (*n* = 3) and electrodes (*n* = 5) were revised in seven patients due to infection.

**Conclusions:**

Pallidal DBS is a safe and efficacious long-term treatment for dystonia with sustained effects on motor impairment and disability, accompanied by a robust improvement in mood and quality of life.

**Electronic supplementary material:**

The online version of this article (10.1007/s00415-020-09745-z) contains supplementary material, which is available to authorized users.

## Introduction

Isolated dystonia is a movement disorder characterized by involuntary sustained or intermittent muscle contractions causing twisting movements and abnormal postures that may be tremulous [1, 2]. Dystonia is distinguished by age of onset, body distribution, temporal pattern, and association with other features [1]. While focal and segmental dystonia are mainly adult onset diseases, generalized dystonia often begins during childhood [1]. Dystonia exerts a relevant influence on everyday life and social participation by combining motor impairment, pain, and social withdrawal [3]. Deep brain stimulation (DBS) of the globus pallidus internus (GPi) is an effective treatment for medically refractory dystonia and reduces not only motor impairment, but also disability [4]. DBS has progressively evolved into a widely available therapeutic strategy in dystonia and is meanwhile applied in patients with complex cervical dystonia or those resistant to botulinum toxin treatment [5]. Therefore, long-term outcome as well as side effects of this potentially lifelong therapy is of special clinical interest. So far, there have been only two larger prospective clinical trials that report long-term improvements of 58% and 60% after a follow-up period of 3 and 5 years, respectively [6, 7]. Other reports include either smaller sample sizes with heterogeneous etiologies of dystonia for up to 10 years [8, 9] or shorter follow-up periods [10, 11].

The impact of DBS on quality of life (QoL) or mood has rarely been studied [12, 13]. Interestingly, detailed analysis of SF36 in larger cohorts 3 and 5 years after stimulation showed heterogeneous effects of DBS on QoL with stable positive effects on health-related physical domains of the questionnaire, but considerably minor effects on mental subdomains.

The aim of the present study was to evaluate long-term effects of bilateral pallidal DBS on motor symptoms, mood, and quality of life in a cohort of 36 consecutive patients with isolated generalized, segmental, or cervical dystonia operated at Charité Hospital, University Medicine Berlin between 2000 and 2007.

## Patients and methods

### Study design and patient cohort

All charts and videos from patients with dystonia who were operated between 2000 and 2007 at Charité Hospital, University Medicine Berlin were reviewed retrospectively (*n* = 60). Thirty-six patients (22 male; mean age at surgery 49 ± 13 years [mean ± standard deviation], range 20–74 years; mean age of onset 36.3 ± 17 years, range 3–60 years; mean disease duration 12.9 ± 9.9 years, range 2–43 years) meeting the criteria for isolated idiopathic or hereditary dystonia [1, 2] were invited for long-term follow-up (LT-FU). Those not invited presented either with complex or tardive dystonia. For the present study, 19 patients (12 male; mean age at surgery 47.3 ± 12 years, range 20–74 years; mean disease duration 13.7 ± 10.9 years, range 2–43 years; mean age of onset 33.1 ± 15 years, range 3–60 years) with isolated generalized (*n* = 10), segmental (*n* = 4) or cervical dystonia (*n* = 5) and chronic pallidal DBS for up to 16 years (mean: 11 ± 2.6 years; range 8–16 years) were examined for LT-FU. None of the patients had any structural brain abnormalities in MRI or other abnormalities contradicting the diagnosis of isolated dystonia. Three patients had a mutation in *TOR1A* (patients 4, 7, 10). Patient 5 had a *THAP1* gene mutation. Three patients (patients 1, 9 and 13) initially received bilateral pallidal and thalamic electrodes (thalamic electrodes were not used in LT-DBS). Assessment at LT-FU (mean 135.3 ± 7.8 months; range 93–197 months) included motor impairment, side effects of stimulation, medication intake, QoL and mood. These data were compared with retrospective data from baseline (preoperative) and short-term follow-up (ST-FU; mean 22.9 ± 4.1 months; range 3–36 months) including video documentation of each visit (for details see Supplementary Table 1). The remaining 17 patients with isolated dystonia that were lost to follow-up had similar demographic characteristics compared to the patients involved in the long-term study (mean age at surgery 50 years (range 26–70), age at onset 36.7 years (range 7–60), a mean baseline TWSTRS in CD of 21.5 points (range 17–28) and a mean BFMDRS score in generalized dystonia of 47.1 points (range 12–89); all not significantly different between groups). These patients were lost to follow-up for various reasons: four patients underwent electrode implantation in our center, but regular follow-up visits were conducted at the individual referral center near patients’ residences immediately following implantation; two patients were lost to follow-up for long-term evaluation due to relocation to other cities; six patients did not consent to follow-up or appeared for follow-ups only irregularly including two patients with *THAP1* gene mutations that did not respond and their DBS system was switched off in the meantime. Five patients were already deceased by the time of long-term follow-up. While three patients died of natural causes, one patient died in consequence of intracerebral bleeding following lysis therapy due to post-operative systemic pulmonary embolism two days after bilateral pallidal lead re-implantation. The fifth patient committed suicide 2 years after bilateral pallidal stimulation.

The study was approved by the local ethics committee and all patients gave their written informed consent for long-term follow-up.

### Surgical technique and device programming

All patients underwent bilateral DBS in the globus pallidus internus (GPi) by the same neurosurgeon (GHS) at the Charité University Hospital, Berlin, using quadripolar electrodes (model 3387 except for patients 4, 12, 14–17 and 19, who received model 3389; Medtronic, Minneapolis, MN, USA). The surgical technique has been reported elsewhere [14]. Electrodes were targeted at the posteroventrolateral portion of the GPi. Patients’ settings were initially programmed within a week after surgery with monopolar settings using 130 Hz stimulation with pulse widths of 90 µs at the contact that had the lowest threshold for phosphenes, but allowed significant stimulation amplitude (contact 0 or 1). Further adjustment was performed at 3 or 6 months after surgery. Patients were seen regularly in the outpatient clinic at intervals of 6–12 months.

### Motor and disability assessment

Dystonia severity and disability were assessed using the Burke–Fahn–Marsden Dystonia Rating Scale (BFMDRS) [15]. For a more detailed analysis of DBS effects on affected body regions, we subdivided the cohort into a subgroup of generalized dystonia patients (*n* = 10) and cervical/segmental dystonia patients (*n* = 9).

In patients with cervical and segmental dystonia, motor impairment and disability were additionally evaluated by means of the Toronto Western Spasmodic Torticollis Rating Scale (TWSTRS) and Tsui rating scale [16, 17]. The scores were also used in patients with segmental dystonia as their predominant motor symptom was cervical dystonia with often only mild involvement of the shoulder or upper extremity. Patients were videotaped according to a standardized protocol at BL and FU visits. All videotapes were rated for motor scores by the same experienced movement disorder experts (PK, AAK). In patients with generalized dystonia, BFMDRS motor scores were further analyzed separately for three subscores: craniocervical (section A–D), trunk (section F) and extremities (sections E and G). We identified non-responders according to the definition used previously as those with a treatment response below 25% [5]. Patients with < 35% motor benefit were defined as poor responders.

### Assessment of mood and quality of life

The individual effects of pallidal stimulation on health-related quality of life and mood were assessed using the SF36 [18] and the BDI [19] at the last follow-up and compared with archival BL data where available.

### Assessment of long-term safety

All reported device-related side effects and adverse events were collected retrospectively from the clinical neurological and neurosurgical records, and patients were asked about chronic side effects or adverse events at LT-FU. Regular exchange of the neurostimulation device due to depletion of the battery was not considered as adverse event.

### Localization of DBS electrodes

**Fig. 1:**
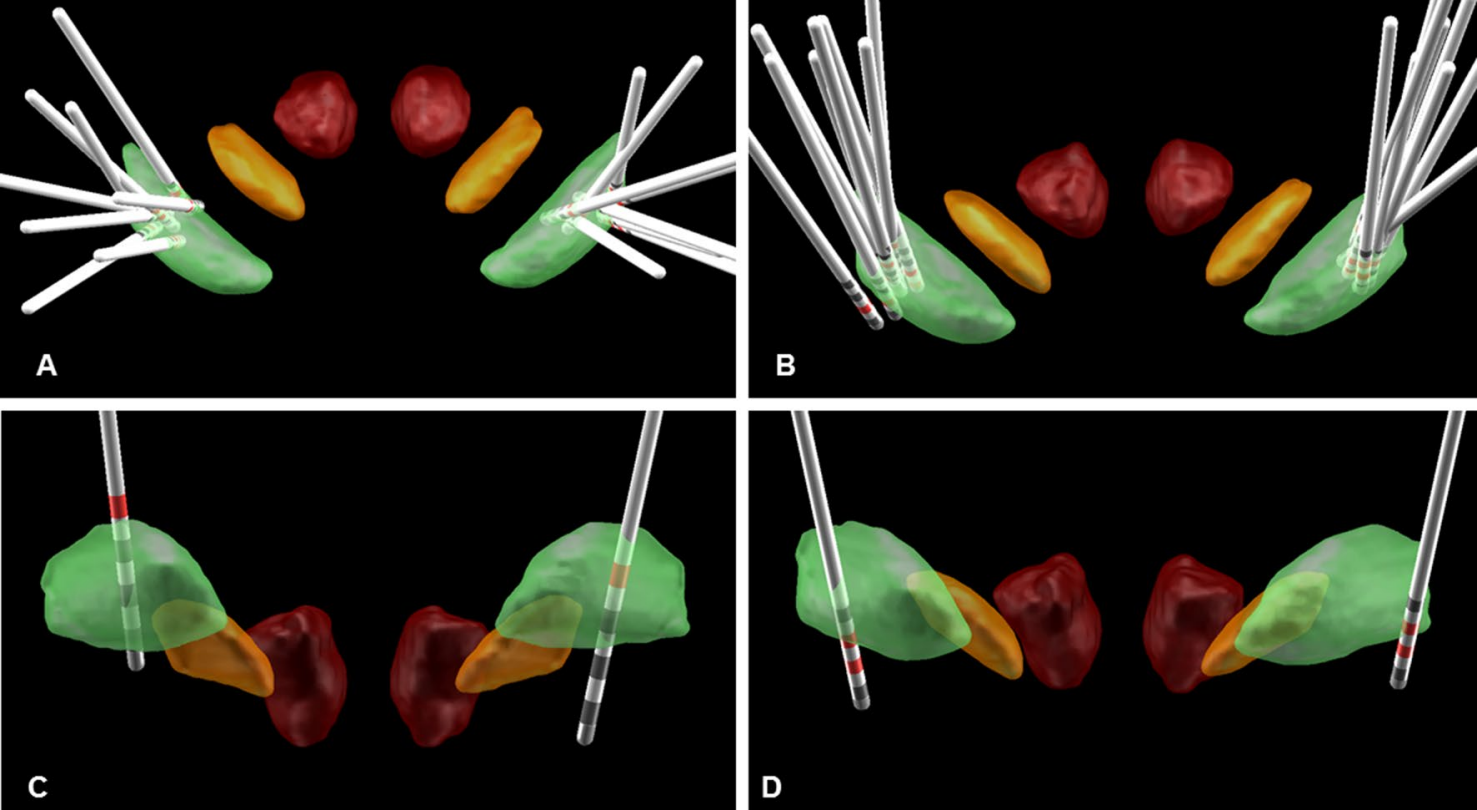
**a**, **b** 3D group visualization of pallidal electrode locations in MNI space highlighting the active contacts (red) in 17 out of 19 patients using the atlas as described in Ewert et al. [25]. Anatomical structures as defined: internal pallidum (green), subthalamic nucleus (orange), and red nucleus (red). **a** Electrode localization of the patients with generalized dystonia (*n* = 8; patients 1 and 9 had to be excluded due to missing preoperative MRI data). **b** Localization analysis of the CD/SD patients (*n* = 9). Electrode localization of patient 2 (**c**) and 4 (**d**) with poor DBS effects. Electrodes are localized within the Gpi. However, clinical choice of active contacts does not perfectly match the visualized boundaries of Gpi. Consequent stimulation adjustments have been successfully initiated (data not shown, here)

DBS electrodes were localized using Lead-DBS ([20]; www.lead-dbs.org) in 17 of 19 patients (Fig. 1; patients 1 and 9 had to be excluded due to missing preoperative MRI data). Postoperative images were co-registered to preoperative MRI using SPM12 (https://www.fil.ion.usl.ac.uk/spm/software/spm12). Pre- and post-operative acquisitions were spatially normalized into MNI ICBM 2009b NLIN ASYM space based on preoperative acquisition(s) (T1 and T2) using the SyN registration approach as implemented in advanced normalization tools ([21]; https://stnava.github.io/ANTs/). 3D visualization (Fig. 1) of group results is shown in MNI space using the atlas as described previously [22].

### Statistical analysis

Motor scores (BFMDRS, TWSTRS, Tsui) and subscores were not normally distributed (Shapiro–Wilk Test, *p* < 0.05); therefore, non-parametric statistics (Friedman test and post-hoc Wilcoxon test) were used for the main analysis on motor improvement. The Mann–Whitney *U* test was used to compare differences between motor improvements in generalized and cervical dystonia. Post-hoc testing between time points in normally distributed data (disability in generalized dystonia, BDI and SF36) was performed using a paired Student's *t* test. Spearman’s correlation was used to investigate the relation of demographic factors such as age at onset and disease severity to DBS motor outcome as well as possible correlations between motor improvement and changes in QoL and mood (SF36 and BDI).

All data are given as mean ± SE, if not mentioned otherwise. A *p *value < 0.05 was considered significant.

## Results

### Clinical improvement

**Fig. 2:**
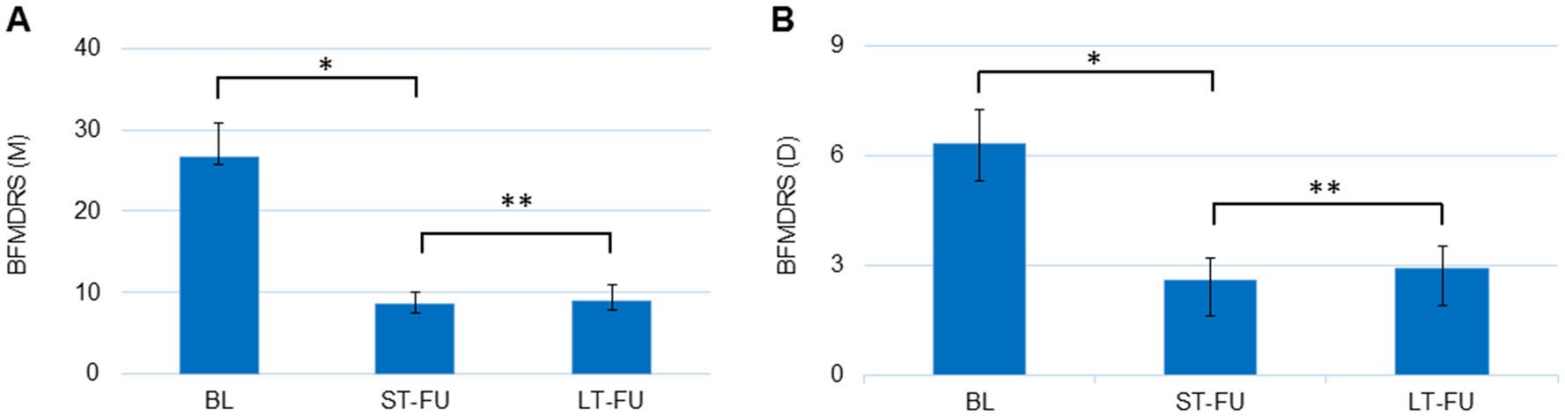
**a**, **b** Mean absolute BFMDRS motor and disability score at baseline, short-term FU (ST-FU) and last long-term FU (LT-FU) of the whole cohort. BFMDRS(M): *p = 0.0002, **p = 0.0001. BFMDRS(D): *p = 0.0006, **p = 0.0006

Motor improvement in the BFMDRS reached 63.8% ± 5.7 at ST-FU (range 25–92) and 67.9% ± 6.1 at LT-FU (range 23–96). Mean disability scores improved by 54.2% ± 9.4 at ST-FU (range 0–100) and 53.8% ± 5.6 at LT-FU (range 0–100). Figure 2 shows the mean motor and disability scores (BFMDRS) at BL, ST-FU, and LT-FU. None of the 19 patients in our cohort were non-responders either at ST-FU or at LT-FU. However, four patients (2 GD and 2 CD) were poor responders. Two of these patients improved at LT-FU due to change of stimulation settings (patient 16 and 19). Patient 2 and 4 remained poor responders with predominant craniocervical and truncal involvement.

#### Generalized dystonia

**Fig. 3:**
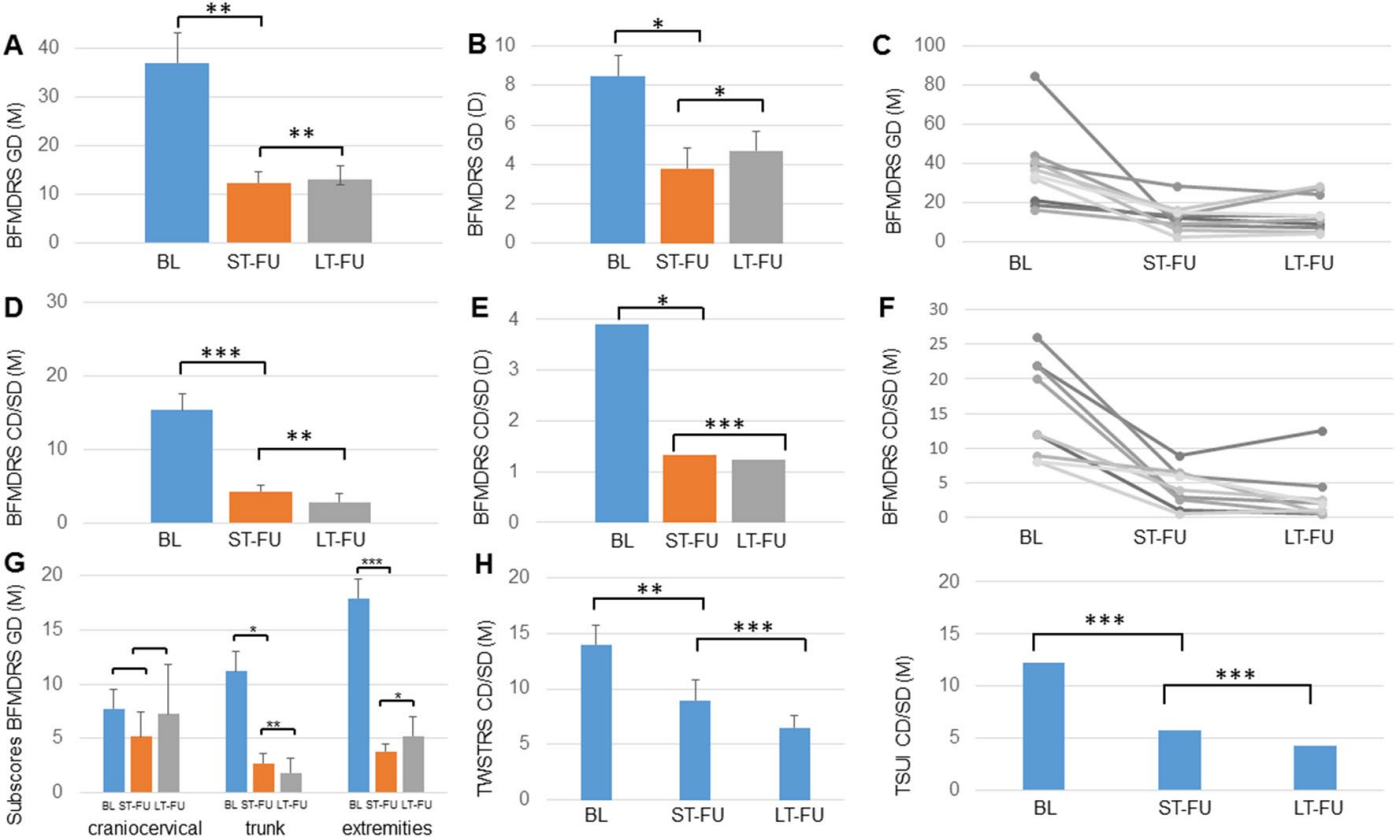
**a**, **b** Mean absolute BFMDRS motor (M) and disability (D) score at baseline (BL), short-term FU (ST-FU) and last long-term FU (LT-FU) of the GD cohort (*n* = 10). **c** Individual absolute BFMDRS motor score at BL, ST-FU and LT-FU of the GD cohort. **d**, **e** Mean absolute BFMDRS motor and disability score at BL, ST-FU and LT-FU of the CD/SD cohort (*n* = 9). **f** Individual absolute BFMDRS motor score at BL, ST-FU and LT-FU of the CD cohort. **g** Mean absolute change in motor subscores for the craniocervical region, trunk and extremities at BL, ST-FU and LT-FU of the GD cohort. Note the significant improvement at ST-FU and LT-FU compared to BL for extremities and trunk. **h** Mean absolute TWSTRS and TSUI score at BL, ST-FU and LT-FU of the CD cohort. *p < 0.05; **p < 0.01; ***p < 0.001

All 10 patients with GD presented with a mean preoperative BFMDR motor score of 36.9 ± 6.2 (range 16–85). The mean relative improvement was 59.7% ± 7.6 at ST-FU (range 28–94%; mean motor BFMDRS ST-FU: 12.4 ± 2.3; range 2–28.5; *p* = 0.005) and 54.4% ± 8.5 at LT-FU (range 28–92%; mean motor BFMDRS LT-FU: 14.3 ± 2.9; range 4–28.5; *p* = 0.006; Fig. 3). Disability was reduced by 46.3% ± 10.5 at ST-FU (range 0–89%; mean disability BFMDRS ST-FU: 3.8 ± 0.8; range 1–9; *p* = 0.012) and by 37.9% ± 10.6 at LT-FU (range 0–94%; mean disability BFMDRS LT-FU: 4.7 ± 0.9; range 1–8; *p* = 0.029).

Motor subscores for the craniocervical region, trunk, and extremities showed significant improvement for the subscores “extremities” and “trunk” (Friedman test, *p* = 0.001), but not for the craniocervical region (mainly speech and swallowing). Post-hoc comparison revealed a significant improvement at ST-FU and LT-FU compared to BL for extremities (ST-FU 77.8% ± 6.4; *p* < 0.001, LT-FU 60.9% ± 6.5; *p* = 0.012) and trunk (ST-FU 70.4% ± 10.3; *p* = 0.012, LT-FU 83.8% ± 5.6; *p* = 0.008; Fig. 3).

#### Cervical and segmental dystonia

The nine patients with CD/SD initially presented with a motor BFMDRS of 15.4 ± 2.2 (range 8–26). Motor impairment improved significantly by 68% ± 8.8 at ST-FU (range 25–94%; mean motor BFMDRS ST-FU: 4.3 ± 0.9; range 0.5–9; *p* = 0.001) and by 83% ± 5.6 at LT-FU (range 43–96%; mean motor BFMDRS LT-FU: 2.9 ± 1.2; range 0.5–12.5; *p* = 0.003). Disability scores decreased by 63% ± 16.1 at ST-FU (range 0–100%; mean disability BFMDRS ST-FU: 1.3 ± 0.6; range 0–5; *p* = 0.009) and by 71% ± 13.8 at LT-FU (range 0–100%; mean disability BFMDRS LT-FU: 1 ± 0.4; range 0–3; *p* = 0.001). Patient 13 reported no benefit in disability due to constant tremor despite of major improvement of dystonia.

Using the more specific rating scores for CD, baseline rating of the motor part (part I, questions A-F) of the TWSTRS was 14 ± 1.8 (range 14–26). Patients showed a significant improvement of 46% ± 11.1 at ST-FU (mean motor TWSTRS at ST-FU: 9; range 1–17; *p* = 0.002) that improved up to 59% ± 5.6 after long-term stimulation (mean motor TWSTRS at LT-FU: 6.5; range 4–14; *p* = 0.0002). Tsui values were reduced about 53% ± 5.9 at short-term and 64% ± 6.9 at LT-FU (mean Tsui at BL: 12; range 6–22; ST-FU: 6; range 3–14; LT-FU: 4; range 1–9; *p* < 0.001). Figure 3 presents individual motor improvements of the cervical/segmental subgroup.

### Correlation of DBS outcome with demographic data

No significant correlation between ages at onset or disease duration with motor outcome was found in our cohorts. Patients with CD had a significantly shorter disease duration (7.7 ± 2 years) at the time of surgery compared to the GD subgroup (19 ± 3.7 years).

### Electrode localization and stimulation parameters

Thirty-eight pallidal electrodes were implanted in 19 patients, and additional 6 thalamic electrodes in 3 patients (with initial quadripolar stimulation). Figure 1 shows a 3D group visualization of pallidal electrode locations in MNI space highlighting the active contacts for each patient. All electrodes were localized with at least one contact within GPi except for the left electrode of patient 8.

The majority of patients had monopolar single or double contact stimulation at ST-FU. The parameter settings were similar at LT-FU in 12 patients with identical choice of active contacts over time and similar parameter settings. Three patients (1, 8, and 10) presented more complex settings at LT-FU compared to ST-FU. Stimulation had been simplified over time in another three patients (12–13, 15). Mean stimulation frequency, pulse width, and amplitude was similar at ST-FU and LT-FU (for details see suppl. Table 2). In six patients (2, 4, 12, 14–16), low frequency stimulation was attempted to further optimize the DBS effects and resulted in improved outcome in two patients (4, 16). No difference in stimulation amplitude was found between patients with generalized and cervical/segmental dystonia (3.16 ± 0.28 V and 3.09 ± 0.27 V) at LT-FU.

### Medication

The number of patients still receiving anti-dystonic medication (e.g. anticholinergics, boutlinumtoxine, and painkillers) at LT-FU had decreased from 17 to 9 (42%). From the remaining eight patients with symptomatic medication additionally to DBS, six patients presented with reduced dosages at LT-FU (see supplementary Table 1).

### Improvement in mood and QoL

#### Mood

Preoperative BDI score was available from 14 of 19 patients. Mean BDI score was 11.7 ± 2.0 (range 2–21) at baseline and was significantly reduced to 6.7 ± 1.5 (range 0–16) at LT-FU amounting to a significant reduction in BDI scores of 37.4% + 12.5 at LT-FU (*p* = 0.007). At LT-FU, two patients fulfilled the criteria for mild depression. Changes in BDI did not correlate with motor improvements.

#### Quality of life

**Fig. 4:**
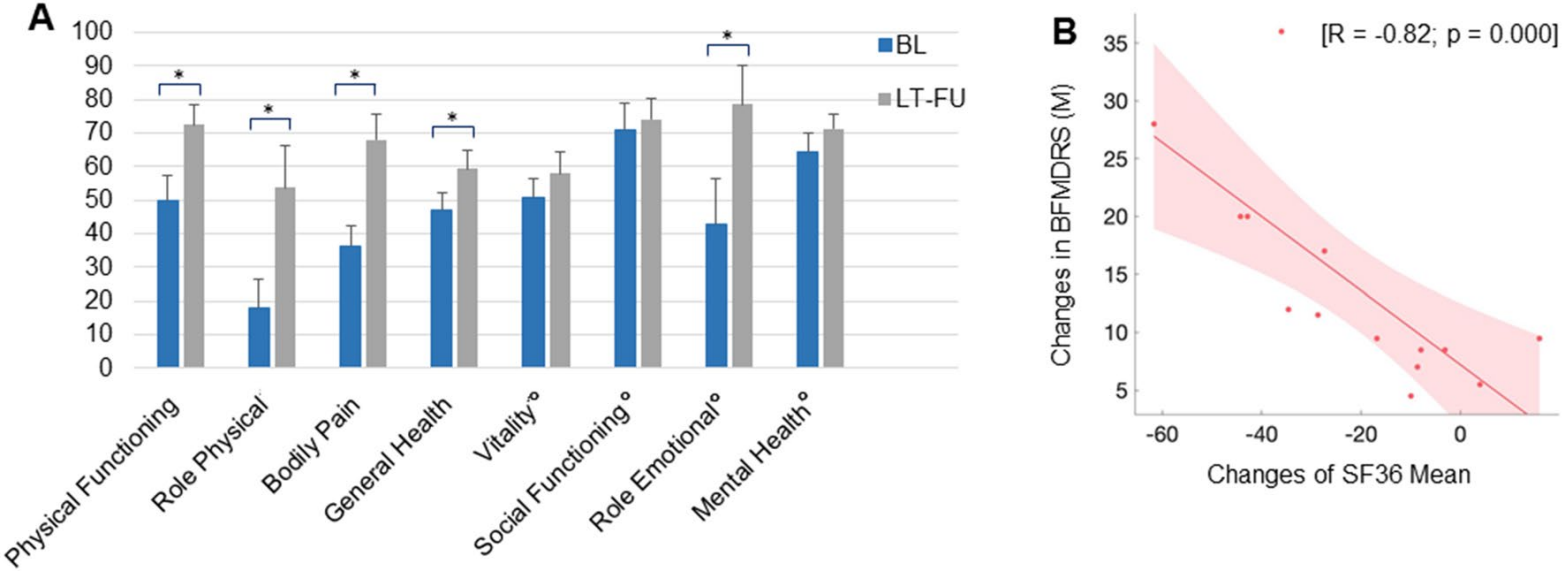
**a** Improvements of quality of life validated by the short form 36 (SF36) at baseline and long-term follow-up. Scores were available from 14 of the 19 patients (*n* = 7 [GD] and *n* = 7 [CD/SD]). Compared to baseline, QoL had ameliorated significantly in all areas of the SF36-physical component summary (PCS) and in the subitem ‘role emotional’ of the SF36-mental component summary (MCS) at LT-FU. Subitems of MCS are indicated by °. 100 points correspond to the maximal degree of QoL. **b** Correlation between improvement of QoL and motor improvement with long-term DBS in absolute numbers. *p < 0.05

Preoperative QoL ratings (SF36) were available from 14 patients showing a significant improvement of 23% of the overall score at LT-FU (67.5 ± 2.6; range 0–100) in comparison to baseline values (45.2 ± 2.9; range 0–100; *p* < 0.001). All subscores of the SF36 physical component score (PCS) reached a significant improvement: 23.5% (*p* = 0.027) in ‘physical functioning’, 41% (*p* = 0.021) in ‘role physical’, 36.3% (*p* = 0.0005) in ‘bodily pain’ and 13.2% (*p* = 0.018) in ‘general health’. However, significant improvements of the SF36 mental component score (MCS) were observed in the subscore ‘role emotional’ (35.7%; *p* = 0.019), but not in the items ‘vitality’, ‘social functioning’, and ´mental health’ (Fig. 4a; for detailed scores see Table 1).

**Table 1:** Improvements of mood and quality of life validated by the Beck depression inventory score (BDI) and Short form 36 (SF36) at baseline and long-term follow-up

Questionnaire	Score range	Mean (SE)	Mean (SE)	*p* value (pre DBS versus LT-FU)
Beck depression inventory score	0–63	11.7 (± 2.0)	6.7 (± 1.5)	0.0066
Short form 36				
Physical functioning	0–100	50 (± 7.2)	73.2 (± 6.0)	0.0269
Role physical	0–100	17.9 (± 8.5)	58.9 (± 12.7)	0.0209
Bodily pain	0–100	36.1 (± 6.3)	72.4 (± 7.5)	0.0005
General health	0–100	47 (± 5.0)	60.2 (± 5.5)	0.0017
Vitality	0–100	50.7 (± 5.7)	59.3 (± 6.3)	0.2853
Social functioning	0–100	70.8 (± 7.9)	76.8 (± 6.1)	0.5159
Role emotional	0–100	42.9 (± 13.7)	78.6 (± 11.4)	0.0186
Mental health	0–100	64.5 (± 5.4)	72.9 (± 4.3)	0.1105

Changes in QoL were associated with motor improvements, shown by a significant negative correlation between changes in the SF36 and changes in the BFMDRS for all patients with available ratings (*r* = − 0.82; *p* < 0.001; Fig. 4b).

### Safety

#### Pulse generator replacement

The main reason for surgical intervention after DBS was the replacement of the pulse generator (IPG) after battery exemption with on average 3.6 ± 3.6 IPG replacements (range 2–8) per patient over a period of 146 months (range 113–190). The mean replacement interval (IPG life span) was 34.9 ± 2.2 months (range 6–72) with 61 IPGs replaced in 19 patients. In one patient, the left GPi electrode had to be removed and reimplanted after intraoperative lesioning during IPG system change. No additional complications, malfunctions, or reduced stimulation effects were observed after IPG changes. Changes to rechargeable IPGs have been discussed with each patient after introduction on the market and with regard to individual replacement interval in each patient.

#### Adverse events and long-term complications

Over the study period, 20 adverse events (AE) were observed in 17 patients. Eight of the 20 adverse events were device related and rated as serious (SAE) requiring hospital admission and surgical intervention (see Table 2). The most common stimulation-related side effects were dysarthria (*n* = 4), swallowing difficulties (*n* = 1) and bradykinesia (*n* = 2), which were all partially reversible with adjustment of stimulation settings. The profile of SAE and side effects did not differ between generalized or cervical/segmental dystonia.

**Table 2:** Adverse events and long-term complications

Adverse events	Number of events	Number of patients
Related to surgery and device		
Wound healing complications	5	3
DBS system revision due to		
Infection of DBS system	3	3
IPG infection	2	2
Lead breakage	1	1
IPG revision due to open circuit	1	1
Electrode migration	1	1
Related to stimulation		
Dysarthria	4	4
Bradykinesia	2	2
Swallowing difficulties	1	1

*SAE* serious adverse event

## Discussion

We herewith present results of long-term follow-up for 8 years and beyond in a group of 19 patients with hereditary or idiopathic forms of isolated dystonia with bilateral pallidal neurostimulation. This study describes sustained improvements of 70% in motor symptoms in dystonia for up to 16 years. Importantly, motor improvement was accompanied by a significant positive long-term impact on QoL and mood in our patients.

### Motor outcome

We show a sustained and highly beneficial long-term motor effect of DBS with about 70% reduction in BFMDRS in our patients that was similar for the groups of cervical/segmental and generalized isolated dystonia. At LT-FU, no patient presented with new symptoms in primarily unaffected body regions as observed in Cif et al. [23, 24]. Overall, our results corroborate those available from previous studies with shorter observation time [7, 25].

Our findings demonstrate significant and enduring stimulation effects in genetic and non-genetic isolated dystonia. Several factors such as gene mutation status, age at surgery, disease duration, presence of musculoskeletal deformities, predominance of phasic versus tonic movements, size of the globus pallidus, and optimal stimulation parameters are widely discussed as possibly influencing stimulation effects [26, 27]. In our cohort, no statistically significant correlation between these clinical parameters and overall DBS improvements was identified.

Electrode localization is a core prerequisite for optimal motor response. We were able to verify optimal lead placement in all but one hemisphere in a single patient from our cohort. Interestingly, the latter patient had a moderate motor improvement, whereas two patients with satisfactory lead placement within the pallidum had a poor motor response. Recent studies suggest that within the pallidum “sweet spots” and “sour spots” could be intermingled leading to suboptimal response when both are activated [28].

In patients with generalized dystonia, subitem analyses revealed a higher benefit for extremities and trunk as compared to the craniocervical region including speech and swallowing. This is in line with previous studies pointing to reduced motor responses for patients with more severe axial symptoms [29]. Moreover, craniocervical symptoms deteriorated by 26% in the GD cohort despite an initial benefit at ST-FU. We cannot but speculate whether the significant and stable benefits on the subitems ‘extremities’ and ‘trunk’ in GD have been achieved to the disadvantage of craniocervical symptoms. The pattern might be related to pallidal anatomy. Former studies in primates demonstrated a somatotopic subdivision of the GPi with the arrangement of face and arm posterior and ventral, and the leg central and more dorsal [30]. Tisch et al. also showed superior efficacy of posteroventral stimulation for upper extremities in patients with generalized dystonia and DBS, while anterodorsal stimulation was best for the leg [31].

In our cohort of CD patients, improvement was less evident in the TWSTRS score (59% ± 5.6) as compared to the relative improvement in BFMDRS (83% ± 5.6) at LT-FU. Nevertheless, overall improvement was slightly higher compared to previous double- and single-blinded studies documenting a respective 26% and 43% reduction of the TWSTRS in patients with CD following DBS [5, 32] being attributed to rating-specific features. It is worth noting that stimulation in our CD subgroup led to additional benefits over time even after the first year of neurostimulation as reported by other groups [11, 33], and which persisted at LT-FU.

### Quality of life

The long-term reduction of motor impairment was paralleled by a significant reduction in depressive symptoms and increased QoL in our cohort. Previous studies not only revealed inconsistent findings for DBS effects on mood and QoL with improved short-term outcome in generalized and CD patients [6, 7, 11–13, 35, 36], but also a lack of improvement in QoL despite motor benefit [35]. In our cohort, we could show that all subitems of the SF36-PCS representing physical aspects of QoL significantly improved with long-term DBS for up to 16 years. In contrast, mental subscores such as ‘mental health’, ‘social functioning’ as well as ‘vitality’ did not improve significantly. Vidailhet and Volkmann [6, 7] observed comparable improvements in physical subdomains of the SF36 with less improvement in mental subdomains emphasizing the need for more data to determine the lack of social functioning in dystonia. Worth noting is that no subitem worsened at LT-FU compared to baseline values.

Strongly related to QoL, patients with dystonia often state reduced self-esteem, a feeling of stigmatization, and they can present with strikingly reduced mental health scores compared to the general population [3]. Depression is increasingly discussed as a non-motor symptom in dystonia [36, 37]. While several groups presented unchanged depression scores pre- and postoperatively [11, 36], motor improvement translated into significant amelioration of mood in studies with longer observation periods [6, 7, 13], which is consistent with our findings. Improvement in QoL was related to the degree of motor improvement here. However, as physical factors and not social functioning improved in the ratings, QoL might be more related to motor items. In line, BDI improvement was unrelated to motor performance either.

### Adverse events

Stimulation-related adverse events occurred in 37% of all patients, comparable to prior studies [5–7]. All stimulation-related side effects were amenable to changes of individual settings. Hardware-related events have been described in up to 25% of patients with DBS [38]. Infection rate associated with DBS after 3 years is reported to range between 0 and 15% with higher rates in children [23, 39]. Long-term infection rate in our cohort was 26% including three system infections and two IPG infections in altogether five patients during a maximum of 16 years of observation. Reimplantation resulted in meaningful symptom reduction in all cases.

## Limitations, strengths, and conclusion

Limitations of this study are the retrospective design with a cohort that was restricted to those patients still visiting our outpatient clinic for LT-FU, the lack of blinded rating and a certain heterogeneity of the cohort with the subgroups of CD and GD.

However, this study presents by far the longest follow-up time of a patient cohort with isolated dystonia and pallidal stimulation of a single center operated by the same neurosurgeon. The main message of this study is the safe and robust effect of DBS on motor impairment and disability for up to 16 years in patients with generalized, segmental, and cervical dystonia that was accompanied by sustaining and significant improvements in mood and quality of life.

This will be even more important as dystonia is a chronic disease of mainly young patients with a regular life expectancy and lifelong need for therapy.

## Electronic supplementary material

Below is the link to the electronic supplementary material.

Supplementary file1 Suppl. Table 1: Demographic characteristics and clinical data (mean ± standard error) of the DBS patients with generalized dystonia (1-10) and cervical/segmental dystonia (11-19). None of the patients had any structural brain abnormalities in individual MRI. Note the complete withdrawal of medication after DBS in 10 of 19 patients. BFMDRS Burke–Fahn–Marsden Dystonia Scale for motor impairment (M) and degree of disability (D) at baseline (BL), short-term follow-up (ST-FU) and last long-term follow-up (LT-FU). p.d.: per day; i.r.: if required; *indicates patients that had been included in the national dystonia trial [7, 40] (DOCX 19 kb)

Supplementary file2 Suppl. Table 2: Stimulation parameters of all patients at short-term and long-term follow-up. Patient numbers given here match table 1 with demographic data of all patients. Patient 18 initially was stimulated quadripolar (Vim and Gpi). After lack of benefit of thalamic stimulation, only bilateral pallidal stimulation was selected (DOCX 20 kb)

Supplementary file3 Suppl. Table 3: Overview of the Drop Outs (n=17; 13 CD/SD versus 4 GD patients) and their individual preoperative and short-term (post DBS) scoresavailable retrospectively (range of ST-FU: 3-60months). * Indicate patients with THAP1 gene mutation. Maximum Value in points for TWSTRS=35,TSUI=25, BFMDRS=120. n. a. = not available (DOCX 20 kb)
