# Correction to: Long-term effects of bilateral pallidal deep brain stimulation in dystonia: a follow-up between 8 and 16 years

**DOI:** 10.1007/s00415-021-10863-5

**Published:** 2021-11-15

**Authors:** P. Krause, S. Völzmann, S. Ewert, A. Kupsch, G. H. Schneider, Andrea A. Kühn

**Affiliations:** 1 Movement Disorder and Neuromodulation Unit, Department of Neurology, Charité, University Medicine Berlin, Campus Mitte, Charitéplatz 1, 10117 Berlin, Germany; 2 Department of Neurosurgery, Charité, University Medicine Berlin, Campus Mitte, Berlin, Germany; 3 Department of Neurology and Stereotactic Neurosurgery, University Medicine of Magdeburg, Magdeburg, Germany

## Correction to: Journal of Neurology (2020) 267:1622–1631 10.1007/s00415-020-09745-z

The article Long‑term effects of bilateral pallidal deep brain stimulation in dystonia: a follow‑up between 8 and 16 years, written by P. Krause, S. Völzmann, S. Ewert, A. Kupsch, G. H. Schneider and Andrea A. Kühn, was originally published Online First without Open Access. After publication in volume 267, issue 6, page 1622–1631 the author decided to opt for Open Choice and to make the article an Open Access publication. Therefore, the copyright of the article has been changed to © The Author(s) 2021 and this article is licensed under a Creative Commons Attribution 4.0 International License, which permits use, sharing, adaptation, distribution and reproduction in any medium or format, as long as you give appropriate credit to the original author(s) and the source, provide a link to the Creative Commons licence, and indicate if changes were made. The images or other third party material in this article are included in the article's Creative Commons licence, unless indicated otherwise in a credit line to the material. If material is not included in the article's Creative Commons licence and your intended use is not permitted by statutory regulation or exceeds the permitted use, you will need to obtain permission directly from the copyright holder. To view a copy of this licence, visit http://creativecommons.org/licenses/by/4.0/.

Open Access funding enabled and organized by Projekt DEAL.

The original article has been corrected.

