# Supplementary material for: Long-term effects of bilateral pallidal deep brain stimulation in dystonia: a follow-up between 8 and 16 years
Source: J Neurol. 2020 Feb 13;267(6):1622–31. doi: 10.1007/s00415-020-09745-z (PMC8592956; doi:10.1007/s00415-020-09745-z)
Supplement: Supplementary file 1 — Supplementary file1 Suppl. Table 1: Demographic characteristics and clinical data (mean ± standard error) of the DBS patients with generalized dystonia (1-10) and cervical/segmental dystonia (11-19). None of the patients had any structural brain abnormalities in individual MRI. Note the complete withdrawal of medication after DBS in 10 of 19 patients. BFMDRS Burke–Fahn–Marsden Dystonia Scale for motor impairment (M) and degree of disability (D) at baseline (BL), short-term follow-up (ST-FU) and last long-term follow-up (LT-FU). p.d.: per day; i.r.: if required; *indicates patients that had been included in the national dystonia trial [7, 40] (DOCX 19 kb) [file 415_2020_9745_MOESM1_ESM.docx]

| Pat. | Sex | Gene mutation | Age at Onset (years) | Age at Surgery (years) | Disease Duration before surgery (years) | Short follow-up (months) | Last follow-up (months) | BFMDRS (M) BL (points) | BFMDRS (D) BL (points) | BFMDRS (M) ST-FU (points) | BFMDRS (D) ST-FU (points) | BFMDRS (M) LT-FU (points) | BFMDRS (D) LT-FU (points) | Medication before surgery | Medication at last follow-up | Type of stimulator (initial/ latest) |
| --- | --- | --- | --- | --- | --- | --- | --- | --- | --- | --- | --- | --- | --- | --- | --- | --- |
| 1 | M | None | 39 | 59 | 20 | 11 | 148 | 21 | 6 | 12 | 5 | 9 | 5 | Painkillers, Botulinumtoxine | None | Kinetra/ActivaRC |
| 2 | F | None | 39 | 58 | 19 | 5 | 167 | 19 | 12 | 13 | 4 | 13.5 | 6 | Lisuride 0.8mg p.d. | Lisuride 0.5 p.d. | Kinetra/Kinetra |
| 3 | M | None | 22 | 49 | 17 | 11 | 119 | 85 | 16 | 8.5 | 1 | 7 | 1 | None | None | Kinetra/Kinetra |
| 4 | M | DYT-TOR1A | 13 | 41 | 28 | 34 | 113 | 39,5 | 7 | 28.5 | 7 | 24 | 7 | Clonazepam 1mg p.d.; Trihexyphenidyl 30mg p.d. | Clonazepam 0.5mg p.d.; Trihexyphenidyl 15mg p.d.; Baclofen 75mg p.d. | Kinetra/ActivaPC |
| 5 | M | DYT-THAP1 | 10 | 20 | 10 | 14 | 159 | 44 | 9 | 13 | 1 | 27 | 6 | Trihexyphenidyl 10mg p.d.; Baclofen 60mg p.d. | None | Kinetra/ActivaPC |
| 6 | F | None | 26 | 36 | 10 | 6 | 154 | 16 | 7 | 9 | 4 | 11.5 | 7 | Botulinumtoxine | None | Kinetra/ActivaRC |
| 7* | M | DYT-TOR1A | 28 | 42 | 14 | 12 | 139 | 41 | 4 | 6 | 2 | 5 | 1 | None | None | Kinetra/ActivaPC |
| 8* | F | None | 3 | 46 | 43 | 25 | 197 | 37 | 16 | 16.5 | 9 | 28.5 | 8 | Trihexyphenidyl 6mg p.d.; Nitomane 50mg p.d.; Lorazepam 1.5mg p.d. | None | Kinetra/ActivaPC |
| 9* | F | None | 32 | 34 | 2 | 71 | 190 | 32 | 5 | 2 | 2 | 4 | 2 | Trihexyphenidyl 45mg p.d.; Nitomane 75mg p.d. | None | Kinetra/Activa RC |
| 10 | F | DYT-TOR1A | 19 | 47 | 28 | 70 | 147 | 34 | 3 | 15 | 3 | 13 | 3 | Nitomane 100mg p.d.; Trihexyphenidyl 7.5mg p.d.; Clonazepam 4mg p.d.; | Clonazepam 6mg p.d. | Kinetra/ActivaRC |
| Mean (±SE) |  |  | 24.1±  4,5 | 43.2±  2,7 | 19.1±  1.8 | 25.9±  7.9 | 153±  8.5 | 36.9± 2.9 | 8.5±  0.7 | 12.4±  0.7 | 3.8±  0.4 | 14.3±  2.9 | 4.6±  0.8 |  |  |  |

| Pat. | Sex | Gene mutation | Age at Onset (years) | Age at Surgery (years) | Disease Duration before surgery (years) | Short follow-up (months) | Last follow-up (months) | BFMDRS (M) BL (points) | BFMDRS (D) BL (points) | BFMDRS (M) 1y-FU (points) | BFMDRS (D) 1y-FU (points) | BFMDRS (M) LT-FU (points) | BFMDRS (D) LT-FU (points) | Medication before surgery | Medication at last follow-up | Type of stimulator (initial/ latest) |
| --- | --- | --- | --- | --- | --- | --- | --- | --- | --- | --- | --- | --- | --- | --- | --- | --- |
| 11 | M | None | 60 | 74 | 14 | 18 | 102 | 12 | 5 | 1 | 0 | 0.5 | 1 | Tillidine 30-40° p.d. | Tillidine 20° p.d. | Kinetra/ActivaPC |
| 12 | M | None | 32 | 41 | 9 | 14 | 112 | 9 | 3 | 6.5 | 3 | 0.5 | 0 | Botulinumtoxine A and B (500 U and 16000 U); Ibuprofene i.r. | Botulinumtoxine (150 U) | Kinetra/ActivaRC |
| 13 | M | None | 39 | 40 | 1 | 3 | 93 | 12 | 3 | 4 | 3 | 2.5 | 3 | Primidone up to 375mg p.d. | Primidone up to 165mg p.d. | ActivaRC/ActivaRC |
| 14 | M | None | 30 | 48 | 18 | 3 | 115 | 8 | 5 | 6 | 5 | 2 | 1 | Diazepam 5-10mg p.d.; Propranolol 40mg p.d. | Diazepam 5-10mg p.d.; Propranolol 20mg p.d. | Kinetra/ActivaPC |
| 15 | F | None | 49 | 58 | 9 | 7 | 98 | 8 | 3 | 0.5 | 0 | 1 | 0 | Botulinumtoxine; Clonazepam 2mg p.d.; Propranolol 160mg p.d. | Clonazepam 1mg p.d.; Propranolol 80mg p.d. | Kinetra/Brio(SJM) |
| 16 | F | None | 54 | 57 | 3 | 15 | 103 | 20 | 4 | 2.5 | 0 | 0.5 | 0 | Indomethacin 150mg p.d.; Trihexyphenidyl 15mg p.d.; Lorazepam 0.5mg p.d. | None | Kinetra/ActivaPC |
| 17 | M | None | 50 | 61 | 11 | 30 | 107 | 22 | 3 | 9 | 1 | 12.5 | 3 | Lorazepam 2mg p.d. | Lorazepam 1mg p.d. | Kinetra/ActivaPC |
| 18* | M | None | 43 | 45 | 2 | 22 | 195 | 26 | 3 | 6 | 0 | 4.5 | 0 | Clonazepam 1mg p.d. | None | Kinetra/ActivaPC |
| 19 | M | None | 40 | 42 | 2 | 38 | 112 | 22 | 6 | 3 | 0 | 2 | 1 | Tetrazepam 75mg p.d.; Ibuprofen 800mg p.d. | None | Kinetra/Kinetra |
| Mean (±SE) |  |  | 44.1±  4.7 | 51.8±  7.5 | 7.7±  2.8 | 16.7±  4.0 | 115.2±  2.4 | 15.4± 2.4 | 3.9±  0.2 | 4.3±  0.5 | 1.3±  0.0 | 2.9±  0.4 | 4.3±  0.0 |  |  |  |
