# Supplementary material for: Long-term effects of bilateral pallidal deep brain stimulation in dystonia: a follow-up between 8 and 16 years
Source: J Neurol. 2020 Feb 13;267(6):1622–31. doi: 10.1007/s00415-020-09745-z (PMC8592956; doi:10.1007/s00415-020-09745-z)
Supplement: Supplementary file 2 — Supplementary file2 Suppl. Table 2: Stimulation parameters of all patients at short-term and long-term follow-up. Patient numbers given here match table 1 with demographic data of all patients. Patient 18 initially was stimulated quadripolar (Vim and Gpi). After lack of benefit of thalamic stimulation, only bilateral pallidal stimulation was selected (DOCX 20 kb) [file 415_2020_9745_MOESM2_ESM.docx]

|  | **Short-term follow-up** | | | | **Long-term follow-up** | | | |
| --- | --- | --- | --- | --- | --- | --- | --- | --- |
| **Patient** | **Active contacts**  **(GPi)** | **Amplitude (V)** | **Frequency (Hz)** | **Pulse width (µs)** | **Active contacts** | **Amplitude (V)** | **Frequency (Hz)** | **Pulse width (µs)** |
| **1** | 3-  4- | 2.5  2.5 | 130 | 90 | 1-2-  5-6- | 2.5  1.5 | 130 | 60 |
| **2** | 2-  6- | 4.4  3.9 | 130 | 60 | 3-  7- | 4.4  4.6 | 150 | 90 |
| **3** | 1+2-  5+6- | 6.0  6.0 | 180 | 90 | 1+2-  5+6- | 4.4  5.0 | 180 | 90 |
| **4** | 1-2-  5-6- | 3.0  3.5 | 30 | 180 | 1-2-  5-6- | 3.3  3.7 | 30 | 180 |
| **5** | 2-3-  6-7- | 3.0  3.0 | 180 | 90 | 2-3-  6-7- | 2.6  3.0 | 180 | 90 |
| **6** | 1-2-  5-6- | 2.0  2.5 | 130 | 120 | 1-2-  5-6- | 2.1  2.6 | 130 | 120 |
| **7** | 1-  5- | 3.3  3.1 | 130 | 90 | 1-  5- | 2.3  2.3 | 130 | 90 |
| **8** | 1-  5- | 3.7  3.8 | 140 | 90 | 1-  5-6- | 3.6  3.4 | 180 | 90 |
| **9** | 0-1-  6-7- | 3.8  2.5 | 185 | 90 | 2-3-  8-9- | 2.0  2.0 | 185 | 90 |
| **10** | 1-  6- | 2.7  2.8 | 130 | 210 | 1-  9- | 2.2  1.5 | 125 | 60 |
| **11** | 1-  6- | 2.5  2.5 | 180 | 90 | 1-  6- | 2.7  2.8 | 195 | 90 |
| **12** | 2-  6- | 4.5  4.0 | 130 | 120 | 1-  6- | 5.0  4.0 | 180 | 90 |
| **13** | 1-  5- | 3.0  3.0 | 130 | 90 | 1-  5- | 2.9  2.9 | 130 | 90 |
| **14** | 0-1-  4-5- | 2.3  2.8 | 185 | 90 | 0-1-  4-5- | 2.4  2.0 | 185 | 90 |
| **15** | 1-  5- | 3.4  4.0 | 130 | 90 | 3-  5-6- | 2.3  4.4 | 210 | 87 |
| **16** | 1+2-3-  5+6-7- | 5.0  5.0 | 60 | 60 | 2-  6- | 1.5  2.0 | 60 | 60 |
| **17** | 1-2-  5-6- | 2.2  2.2 | 180 | 90 | 1-  5- | 3.7  4.0 | 130 | 60 |
| **18** | GPi 1-2-  6-7-  Vim 1-2-  6-7- | 2.0  3.5  2.3  1.2 | 130  100 | 90  210 | 1-  9-  OFF | 2.6  2.6  OFF | 130  OFF | 90  OFF |
| **19** | 1-  5- | 3.2  3.2 | 130 | 90 | 1-  5- | 3.0  3.2 | 130 | 90 |
