# Supplementary material for: Long-term effects of bilateral pallidal deep brain stimulation in dystonia: a follow-up between 8 and 16 years
Source: J Neurol. 2020 Feb 13;267(6):1622–31. doi: 10.1007/s00415-020-09745-z (PMC8592956; doi:10.1007/s00415-020-09745-z)
Supplement: Supplementary file 3 — Supplementary file3 Suppl. Table 3: Overview of the Drop Outs (n=17; 13 CD/SD versus 4 GD patients) and their individual preoperative and short-term (post DBS) scoresavailable retrospectively (range of ST-FU: 3-60months). * Indicate patients with THAP1 gene mutation. Maximum Value in points for TWSTRS=35,TSUI=25, BFMDRS=120. n. a. = not available (DOCX 20 kb) [file 415_2020_9745_MOESM3_ESM.docx]

| \| Patient \| Type of Dystonia \| TWSTRS  (pre DBS, points) \| TSUI  (pre DBS,  points) \| BFMDRS  (pre DSB,  points) \| TWSTRS  (ST-FU*,  points) \| TSUI  (ST-FU*,  points) \| BFMDRS  (ST-FU*,  points) \| Remarks \| Non-Responder \| \| --- \| --- \| --- \| --- \| --- \| --- \| --- \| --- \| --- \| --- \| \| 20 \| CD \| 21 \| 22 \| n.a. \| 15 \| 10 \| n.a. \| Irregular FU/No consent \|  \| \| 21 \| CD \| 17 \| n.a. \| n.a. \| 11 \| 5 \| n.a. \| Lost to FU due to relocation \|  \| \| 22 \| CD \| 28 \| 14 \| n.a. \| 4 \| 3 \| n.a. \| Lost to FU due to relocation \|  \| \| 23 \| CD \| 18 \| 13 \| n.a. \| n.a. \| 10 \| n.a. \| Implantation only \|  \| \| 24 \| CD \| 26 \| 22 \| n.a. \| n.a. \| 13 \| n.a. \| Implantation only \|  \| \| 25 \| SD \|  \|  \| 13 \| n.a. \| n.a. \| 6 \| Implantation only \|  \| \| 26 \| SD \| 18 \| 12 \| n.a. \| 13 \| n.a. \| n.a. \| Irregular FU/No consent \|  \| \| 27 \| SD \| n.a. \| n.a. \| 23 \| n.a. \| n.a. \| 3 \| Deceased due to natural cause \|  \| \| 28 \| CD \| 20 \| n.a. \| 31 \| 7 \| n.a. \| 10 \| Committed suicide \|  \| \| 29 \| CD \| n.a. \| 17 \| 22 \| n.a. \| 3.5 \| n.a. \| Deceased due to natural cause \|  \| \| 30 \| CD \| 24 \| 16 \| n.a. \| 19 \| 13 \| n.a. \| Deceased due to malignoma \| X \| \| 31* \| SD \| n.a. \| n.a. \| 12 \| n.a. \| n.a. \| 13 \| Stim OFF, 2006 VoA and also OFF \| X \| \| 32 \| SD \| 22 \| n.a. \| 33 \| 5 \| n.a. \| 6.5 \| Irregular FU/No consent \|  \| \| 33* \| GD \| n.a. \| n.a. \| 89 \| n.a. \| n.a. \| 59 \| Stim OFF \| X \| \| 34 \| GD \| n.a. \| n.a. \| 60.5 \| n.a. \| n.a. \| 65 \| Irregular FU/No consent \| X \| \| 35 \| GD \| n.a. \| n.a. \| 15 \| n.a. \| n.a. \| 5.5 \| Implantation only \|  \| \| 36 \| GD \| 18 \| n.a. \| 23 \| 11 \| n.a. \| 17 \| Died in consequence of lung artery embolism \|  \| |
| --- | --- | --- | --- | --- | --- | --- | --- | --- | --- | --- | --- | --- | --- | --- | --- | --- | --- | --- | --- | --- | --- | --- | --- | --- | --- | --- | --- | --- | --- | --- | --- | --- | --- | --- | --- | --- | --- | --- | --- | --- | --- | --- | --- | --- | --- | --- | --- | --- | --- | --- | --- | --- | --- | --- | --- | --- | --- | --- | --- | --- | --- | --- | --- | --- | --- | --- | --- | --- | --- | --- | --- | --- | --- | --- | --- | --- | --- | --- | --- | --- | --- | --- | --- | --- | --- | --- | --- | --- | --- | --- | --- | --- | --- | --- | --- | --- | --- | --- | --- | --- | --- | --- | --- | --- | --- | --- | --- | --- | --- | --- | --- | --- | --- | --- | --- | --- | --- | --- | --- | --- | --- | --- | --- | --- | --- | --- | --- | --- | --- | --- | --- | --- | --- | --- | --- | --- | --- | --- | --- | --- | --- | --- | --- | --- | --- | --- | --- | --- | --- | --- | --- | --- | --- | --- | --- | --- | --- | --- | --- | --- | --- | --- | --- | --- | --- | --- | --- | --- | --- | --- | --- | --- | --- | --- | --- | --- | --- | --- | --- | --- |

**Suppl. Table 3:** Overview of the Drop Outs (n=17; 13 CD/SD versus 4 GD patients) and their individual preoperative and short-term (post DBS) scores available retrospectively (range of ST-FU: 3-60months). * Indicate patients with *THAP1* gene mutation. Maximum Value in points for TWSTRS=35, TSUI=25, BFMDRS=120. n. a. = not available.
